# Closing the gap? Results-based financing and socio-economic-related inequalities in maternal health outcomes in Zimbabwe

**DOI:** 10.1093/heapol/czae080

**Published:** 2024-08-24

**Authors:** Marshall Makate, Nyasha Mahonye

**Affiliations:** Health Economics and Data Analytics, School of Population Health, Curtin University, GPO Box U1987, Perth, WA 6845, Australia; School of Economics and Finance, University of the Witwatersrand, P Bag 3, Johannesburg 2050, South Africa

**Keywords:** Results-based financing, maternal health outcomes, wealth-related inequality, relative inequality, absolute inequality, extended two-way fixed effects, Zimbabwe

## Abstract

The results-based financing (RBF) programme, first implemented in Zimbabwe in 2011 and gradually expanded to other districts, aimed to address disparities in maternal health outcomes by improving the utilization of health services. This study leverages the staggered rollout of the programme as a quasi-experimental design to assess its impact on asset wealth-related inequalities in selected maternal health outcomes. The objective is to determine whether RBF can effectively reduce these disparities and promote equitable healthcare access. We employ an extended two-way fixed effects (ETWFE) model to exploit temporal variation in RBF implementation as well as individual-level variation in birth timing for identification. Utilizing pooled cross-sectional and nationally representative data from the Zimbabwe demographic and health surveys collected between 1999 and 2015, our analysis reveals significant reductions in relative and absolute maternal health inequalities, especially in the frequency and timing of prenatal care, delivery by caesarean section and family planning. Specifically, the RBF programme is associated with reductions in disparities for completing at least four or more prenatal care visits (−0.026, *P* < 0.01), first-trimester prenatal care (−0.033, *P* < 0.01), delivery by caesarean section (−0.028, *P* < 0.005) and family planning (−0.033, *P* < 0.005). Additionally, the programme is associated with improved prenatal care quality, as evidenced by progress on the prenatal care quality index (−0.040, *P* < 0.01). These effects are more pronounced among lower socio-economic groups in RBF districts, highlighting RBF’s potential to promote equitable healthcare access. Our findings advocate for targeted policy interventions prioritizing expanding access to critical maternal health services in underserved areas and incorporating equity-focused measures within RBF frameworks to ensure inclusive and effective healthcare delivery in Zimbabwe and other low-income countries.

Key messages

- This study uses the staggered rollout of the results-based financing (RBF) programme as a quasi-experimental design, employing an extended two-way fixed effects model to examine how temporal variations in implementation and birth timing affect asset wealth-related inequalities in maternal health outcomes in Zimbabwe.
- Our findings demonstrate that the RBF programme is associated with reduced asset wealth-related disparities in maternal health services, particularly in the frequency and timing of prenatal care, caesarean section deliveries and family planning.
- The programme is associated with significant reductions in asset wealth-related inequalities in prenatal care quality, as indicated by notable improvements in blood pressure checks and tetanus toxoid vaccinations for women from disadvantaged backgrounds.
- These results highlight the importance of integrating equity-focused measures into RBF frameworks and prioritizing access to essential maternal health services in underserved areas to promote inclusive and effective healthcare delivery in Zimbabwe.

## Introduction

As global health systems strive to improve maternal healthcare, results-based financing (RBF) programmes have gained prominence for their potential to align financial incentives with health outcomes. Over the last few decades, RBF programmes have significantly influenced global health economics, particularly in low-income countries (James *et al*., 2020). These programmes hold immense potential for aiding vulnerable groups, especially women and children, by incentivizing healthcare providers to meet performance targets and, in some cases, offering financial incentives to patients for engaging in health-related actions (Eichler and Levine, 2009; Morgan, 2012; World Bank, 2016). The literature highlights RBF’s capacity to reshape health system financing by aligning economic incentives and resource allocation principles to improve the quality and accessibility of maternal healthcare (Witter *et al*., 2019; James *et al*., 2020). This approach reflects Kenneth Arrow’s foundational insights into the unique nature of health markets and the importance of efficiently managing scarce resources to promote health equity, especially in resource-constrained environments (Arrow, 1963). In particular, RBF initiatives in Zimbabwe aim to translate service availability into measurable health outcomes by encouraging providers to deliver critical services such as prenatal care, skilled birth attendance and postnatal follow-up (World Bank, 2016).

Despite the potential of RBF programmes to enhance maternal healthcare access and equity, their effectiveness in low-income countries like Zimbabwe remains uncertain due to insufficient comprehensive evidence on their ability to address socio-economic-related disparities effectively (Mathonnat and Pelissier, 2017). Women from wealthier families disproportionately access maternal healthcare services (Makate and Makate, 2017; Leventhal *et al*., 2021), a disparity exacerbated by user fees and other structural barriers (Gage, 2007; Houweling *et al*., 2007; Creanga *et al*., 2011; Zeng *et al*., 2018). While RBF is associated with increased healthcare utilization, the programmes often fail to distribute benefits evenly, leaving the poorest and most marginalized populations behind (Mathonnat and Pelissier, 2017; Lopez, 2018). For example, integrating RBF with targeted equity measures in Burkina Faso has shown promise in increasing healthcare service use among the ultra-poor, indicating that well-designed interventions can reduce disparities (Mwase *et al*., 2022). However, achieving true equity through RBF remains challenging, with various studies emphasizing the importance of context-specific design, implementation and evaluation (Fox and Reich, 2015; Paul *et al*., 2018). This study looks closely at Zimbabwe’s RBF programme to assess its impact on socio-economic-related disparities in maternal health outcomes. By analysing how RBF influences healthcare access across different socio-economic groups, we aim to generate evidence-based insights to inform new policies and improve existing ones, promoting more equitable health outcomes in resource-constrained settings.

In this paper, we contribute to the literature by leveraging Zimbabwe’s RBF programme as a quasi-experimental approach to examine its effects on asset wealth-related inequalities in maternal health outcomes. We assess whether RBF can promote social equity by effectively reducing asset wealth-based disparities in access to essential maternal healthcare services in Zimbabwe. Our empirical approach employs an extended two-way fixed effects (ETWFE) model (Wooldridge, 2021), a method recently utilized in a related study examining the impact of RBF on inequality of opportunity (IOP) in maternal health outcomes in Zimbabwe (Makate, 2024), to address the staggered implementation of the RBF programme across various districts and periods. This model mitigates biases associated with traditional two-way fixed effects (TWFE) estimators, mainly when treatment effects vary over time and across groups (De Chaisemartin and d’Haultfoeuille, 2020; Callaway and Sant’Anna, 2021; Goodman-Bacon, 2021). We utilize data from the nationally representative Zimbabwe demographic and health survey (DHS) geo-linked to health facility locations to measure each household’s proximity to health facilities. This quasi-experimental design leverages the staggered adoption of RBF, temporal variation in its introduction, and individual-level variation in the timing of birth for identification. Our findings reveal significant reductions in asset wealth-related inequalities in selected maternal health outcomes, including prenatal care, delivery services and prenatal care quality, whether measured in relative [using the concentration index (CI)] or absolute [using the slope index of inequality (SII)] terms. The results underscore RBF’s potential to promote equitable healthcare access in low-income countries like Zimbabwe, providing crucial insights for policymakers and healthcare professionals.

## RBF programme in Zimbabwe

**Figure 1.**
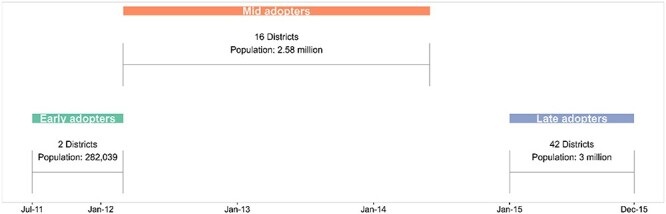
Staggered implementation of the RBF programme in Zimbabwe, showing the classification of districts into early, mid and late adopters based on their programme start dates. Source: Adapted from World Bank (2016)

The Zimbabwean government initiated the RBF programme in July 2011 as a strategic intervention to enhance maternal and child health services (World Bank, 2016). This programme aimed to improve the healthcare system’s efficiency, equity and accountability, marking a significant shift in healthcare delivery and financing in the country (World Bank, 2013). The rollout of the RBF programme occurred in three distinct phases, demonstrating a carefully staggered approach to implementation. The programme began with early adopters in July 2011, launching in two pilot districts: Zvishavane and Marondera. This initial phase allowed for testing and refinement of the RBF model in the Zimbabwean context. The second phase, involving mid-adopters, commenced in March 2012, expanding to 16 additional districts. This substantial scaling up significantly increased the programme’s reach and impact. The third phase, encompassing late adopters, expanded further to the remaining 42 districts from July 2014. This final phase completed the national rollout of the RBF programme, extending its coverage to a broader population across the country. A list of the districts involved in each RBF phase is available in the Supplementary materials. By 2016, the programme had reached ∼3.5 million people across 385 health facilities (World Bank, 2016). Figure 1 summarizes the phased rollout, showing the systematic extension of the RBF programme from the initial districts to a broader population across the country. Funding for the programme came from multiple sources, reflecting a collaborative approach to health financing. The World Bank’s Health Results Innovation Trust Fund provided an initial grant of $15 million, supplemented by co-funding from Zimbabwe’s Ministry of Finance and Economic Development. The programme’s execution was entrusted to the non-governmental organization Cordaid, leveraging their expertise in health system strengthening (World Bank, 2016).

The RBF programme maintained a standardized approach throughout its implementation, focusing on three key elements: result-based contracting, capacity building and thorough monitoring (World Bank, 2016). The Ministry of Child Health identified 16 critical maternal and child health indicators for incentivization, including prenatal care, HIV testing and treatment, and services for children <5 years (World Bank, 2013; 2016). This comprehensive set of indicators ensured that the programme addressed various essential health services. District hospitals received payments based on critical birth-related indicators and a ‘remoteness bonus’ to promote care in underserved communities or regions. The programme design allowed for decentralized decision-making, enabling facilities to reinvest ∼25% of RBF funds for infrastructure and service improvement (World Bank, 2016). The remaining 75% of the funds were intended to be used for direct healthcare service delivery, including covering operational costs and providing incentives for healthcare providers to improve performance and meet specific health targets (Kovacs *et al*., 2022). To improve equity and accessibility, extra incentives were provided to health facilities in remote areas. This multifaceted incentive structure, focusing on service quantity, quality and patient satisfaction, highlighted the programme’s comprehensive approach to healthcare improvement (World Bank, 2016).

## Data and methodology

### Data sources

#### Zimbabwe DHS

The primary data source is the Zimbabwe DHS^1^, a publicly accessible, nationally representative household-level dataset. We focus on data from four DHS rounds collected in 1999, 2005/2006, 2010/2011 and 2015, explicitly extracting information from the individual recode file, which contains detailed health-related data for women aged 15–49 years and their children born within the 5 years preceding each survey. The DHS comprehensively captures a wide array of health indicators, including fertility, family planning, maternal and child health, nutrition and socio-economic factors, offering a comprehensive overview essential for our analysis.

#### Geo-linking the Zimbabwe DHS data to health facility locations

To incorporate spatial dimensions into our analysis, we utilized the geographic data provided by the Zimbabwe DHS, which includes precise longitude and latitude coordinates for each household. These spatial dimensions are particularly important in the Zimbabwean context, where access to healthcare services can vary significantly across different regions. Although these data enable us to match households to primary sampling units (DHS clusters), it does not include information on the second administrative unit, namely districts, where the RBF operates. To address this limitation, we integrated DHS geographic data with administrative data from the Global Administrative Areas (GADM) for Zimbabwe (Global Administrative Areas, 2022). The GADM dataset provides high-resolution maps and data on administrative divisions, including provinces and districts, making it widely used in geographic and demographic research. In the context of Zimbabwe, the GADM data are crucial for accurately mapping and analysing administrative boundaries, enabling the precise geographic linkage required for our study. We used ArcMap version 10.4 to map DHS clusters to their respective districts and subsequently created a district-level panel dataset, capturing information such as cluster number, survey year, province name and number, district name and place of residence (rural or urban). For more details on data processing, refer to the related working paper (Makate and Mahonye, 2023).

Following this data integration, we use the Haversine formula to estimate the straight-line distance (‘as the crow flies’) from each household to the nearest health facility. This method contrasts with that of Milcent (2023), who calculates road distances accounting for actual travel routes. Our straight-line distance estimation provides a different perspective, capturing the geographical accessibility of health facilities, particularly relevant in rural settings where road networks may be less developed or direct. This method, renowned for its accuracy in estimating straight-line distances on the earth’s spherical surface, is widely used in economic studies for measuring geographical proximity due to its precision (Hernæs and Skyrud, 2022). This method enabled us to calculate the distance to the nearest health facilities, which we included as an additional control variable in all our regressions. Accounting for the distance to health facilities remains essential, particularly in remote locations where access challenges can significantly influence the effectiveness of the RBF programme. Understanding these dynamics is crucial for accurately assessing the programme’s impact and designing policies that improve healthcare accessibility.

#### Measuring asset wealth-related inequality in maternal health outcomes

To measure asset wealth-related inequality in maternal health outcomes, we utilize the CI for health, a standard bivariate rank-dependent index widely used in health economics (Kakwani *et al*., 1997; Wagstaff, 2005). The CI assesses an individual’s health relative to their socio-economic status, which is determined in our study by an asset-based household wealth index derived using principal components analysis (Rutstein, 2015). This asset index is pre-calculated in the DHS survey data and is widely used in low-income countries where detailed income data are often limited. Given the binary nature of many maternal health variables, such as prenatal care (e.g. completing four or more visits), different values of measured relative inequality indices can arise (Clarke *et al*., 2002). Erreygers (2009) proposed a corrected CI (CCI) to address limitations of the traditional CI, particularly when dealing with bounded health variables. The traditional CI quantifies the systematic relationship between health and household wealth into an index within the $\left[ { - 1\,,\,1} \right]$ interval. Negative values of this CI indicate that the outcome is more concentrated among poorer individuals, while positive values suggest a concentration among wealthier individuals (Wagstaff, 2005). The CCI, on the other hand, adjusts for the inherent biases present in the traditional CI, providing a more accurate measure of inequality when health variables have natural limits. We use the CCI to measure asset wealth-related inequality in maternal health outcomes for each district $d$ at time $t$ (${\mathrm{MHIne}}{{\mathrm{q}}_{{\mathrm{dt}}}}$). This index can be expressed as follows:


(1) $${\mathrm{MHIne}}{{\mathrm{q}}_{{\mathrm{dt}}}}\left( {{h_{idt}}} \right) = \frac{{4\bar h}}{{{h^{max}} - {h^{min}}}} \times {\mathrm{C}}{{\mathrm{I}}_{{\mathrm{dt}}}}$$

where, ${\mathrm{MHIne}}{{\mathrm{q}}_{{\mathrm{dt}}}}$ is a measure of maternal health inequality outcome (e.g. completion of four or more prenatal care visits) in district $d$ at time $t$, ${h_{idt}}$ is a measure the health outcome of person $i$ living in district $d$ at time $t$, $\bar h$ denotes the mean of the health variable, ${h^{min}}$ and ${h^{max}}$ are the lower and maximum values of the health variable and ${\mathrm{C}}{{\mathrm{I}}_{{\mathrm{dt}}}}$ is the standard or traditional CI, as defined in Kakwani *et al*. (1997).

Table 1 presents weighted summary statistics for women in the 2010 Zimbabwe DHS, highlighting key demographic and socio-economic characteristics across RBF adoption statuses. Overall, early adopters are generally younger, more educated and reside closer to health facilities than mid-adopters, reflecting greater accessibility and educational attainment. They also exhibit lower rates of multidimensional energy poverty and better housing conditions, with improved access to utilities such as piped water. However, health insurance coverage remains low across all groups, and employment is marginally higher among mid-adopters.

**Table 1.** Weighted summary statistics of women in the analysis sample, by RBF adoption status

	Overall sample		Early adopters		Mid-adopters	
Variables	Mean	SD	Mean	SD	Mean	SD
Age (in years) at survey date	28.13	(9.46)	26.93	(9.74)	28.25	(9.42)
Age at first birth	19.84	(3.10)	20.74	(3.30)	19.77	(3.07)
Years of completed schooling	8.76	(3.16)	9.99	(1.95)	8.64	(3.22)
Completed secondary school	0.62	(0.49)	0.84	(0.36)	0.59	(0.49)
Female head of household	0.46	(0.50)	0.31	(0.46)	0.47	(0.50)
Apostolic church membership	0.37	(0.48)	0.38	(0.49)	0.37	(0.48)
Health insurance	0.07	(0.25)	0.10	(0.29)	0.06	(0.25)
Household asset wealth						
Asset quintile 1 (poorest)	0.22	(0.42)	0.03	(0.17)	0.24	(0.43)
Asset quintile 2	0.19	(0.39)	0.24	(0.43)	0.19	(0.39)
Asset quintile 3	0.19	(0.39)	0.22	(0.42)	0.19	(0.39)
Asset quintile 4	0.22	(0.41)	0.25	(0.44)	0.21	(0.41)
Asset quintile 5	0.18	(0.39)	0.25	(0.44)	0.17	(0.38)
	9.05	(14.94)				
Distance (in kilometres) to nearest health facility			2.88	(2.35)	9.63	(15.49)
Multidimensional energy poverty	0.80	(0.40)	0.63	(0.48)	0.82	(0.39)
Improved housing	0.65	(0.48)	0.79	(0.40)	0.63	(0.48)
Piped water	0.56	(0.50)	0.73	(0.45)	0.54	(0.50)
Improved toilet	0.56	(0.50)	0.78	(0.42)	0.54	(0.50)
Currently employed	0.20	(0.40)	0.18	(0.38)	0.21	(0.40)
Urban resident	0.24	(0.43)	0.43	(0.50)	0.22	(0.42)
Observations	2560		184		2376	

Notes: Source: Data are from the 2010/2011 Zimbabwe DHSs.

### Empirical strategy

To estimate the impact of RBF on asset wealth-related inequalities in selected maternal health outcomes, we employ the ETWFE estimator (Wooldridge, 2021). A common approach to estimating the impact of policy interventions is the difference-in-differences (DD) model, which includes a post-treatment period dummy, a treatment indicator and an indicator for eventually being treated. The DD estimator is numerically equivalent to a linear model incorporating both unit and time-fixed effects, similar to ordinary least squares via the TWFE estimator.

However, the staggered implementation of the RBF programme across different districts and times requires an approach that goes beyond the basic TWFE estimator. Recent research has highlighted limitations in the TWFE estimator for staggered interventions, mainly when treatment effects vary across time and groups (De Chaisemartin and d’Haultfoeuille, 2020; Goodman-Bacon, 2021). This research indicates that multiperiod DD estimators can produce biased estimates due to negative weighting, making it challenging to identify the policy intervention’s average treatment effect (ATT) using the standard TWFE estimator. We use the ETWFE estimator that Wooldridge (2021) proposes to address these limitations. This model is better suited for capturing the dynamic treatment effects in a staggered implementation context and applies to repeated cross-sectional data. We define exposure to the RBF programme at the individual level, based on the month and year of conception relative to the treatment start month and year in each district (Fichera *et al*., 2021). Women are classified into treatment cohorts as follows: ${\mathrm{corhot}}2011$—for those who conceived on or after 1 July 2011 (exposed to early RBF), ${\mathrm{corhot}}2012$—for those who conceived on or after 1 March 2012 and ${\mathrm{corhot}}2014$—for those who conceived on or after 1 July 2014.

Our ETWFE model is thus specified as follows:


(2) $$\begin{aligned}{\mathrm{MHIne}}{{\mathrm{q}}_{{\mathrm{dt}}}} = & {\alpha } + \mathop \sum \limits_{r = q}^T \mathop \sum \limits_{s = r}^T {\tau _{rs}}\left( {{w_{idt}} \times {d_r} \times {f_{st}}} \right) + {{\gamma }_t} + {{\delta }_{\mathrm{d}}} + {\phi _c} \nonumber \\& + {X_{idt}}\lambda + { \in _{{\mathrm{idt}}}}\end{aligned}$$

where, ${\mathrm{MHIne}}{{\mathrm{q}}_{{\mathrm{dt}}}}$ measures asset wealth-related inequality in maternal health outcome in district $d$ at time $t$, ${\tau _{rs}}$ are the treatment effect parameters for districts first exposed to the RBF in period $r$ and observed in period $s$, ${d_r}$ is a dummy variable indicating first treatment period for district $d$, ${f_{st}}$ is a time dummy and ${w_{idt}}$ is a time-varying treatment indicator at the individual level, defined by the year and month of conception relative to the RBF start date in their district. The parameters ${{\gamma }_t}$, ${{\delta }_{\mathrm{d}}}$ and ${\phi _c}$ represent time, district and cluster-level fixed effects, respectively. We include time fixed effects, ${{\gamma }_t}$, to control for national-level influences, such as economic trends and policy changes, affecting all districts similarly over time. District fixed effects, ${{\delta }_{\mathrm{d}}}$, account for unobserved, time-invariant characteristics unique to each district, such as geographic or cultural factors that may contribute to differences in maternal health inequality. Cluster fixed effects, ${\phi _c}$, account for within-cluster correlations and unobserved heterogeneity at the cluster level, improving the precision of our estimates. The vector ${X_{idt}}$ captures individual and district-level covariates including distance to the nearest health facility and ${ \in _{{\mathrm{idt}}}}$ is an error term, with standard errors clustered at the district level.

To compute the overall ATT, we aggregate the coefficients of the interaction terms by averaging them across all cohorts and post-treatment periods, following Wooldridge (2021):


(3) $${\mathrm{ATT}} = \frac{1}{N}\mathop \sum \limits_{c \in \left\{ {2011,2012,2014} \right\}} \mathop \sum \limits_{t = 0}^T {\tau _{rs}}$$

where, $N$ is the number of non-collinear interaction coefficient terms. The DD methodology relies on the assumption that, in the absence of the RBF intervention, the trends in asset wealth-related maternal health inequality outcomes would have been similar between RBF and non-RBF districts. The ETWFE estimator offers an effective way to test this parallel trends assumption (Wooldridge, 2021). In our ETWFE regression, we include heterogenous linear trends $\left( {{d_{iq}} \times t, \ldots ,{d_{iT}} \times t} \right)$, where $t$ represents the pre-RBF periods, and conduct a joint significance test to assess whether these interaction terms collectively have a significant effect on the outcomes, indicating whether the trends differ systematically across groups. The equation for testing the parallel trends assumption is specified as follows (Wooldridge, 2021):


$${\mathrm{MHIne}}{{\mathrm{q}}_{{\mathrm{dt}}}} = {\alpha } + \mathop \sum \limits_{r = q}^T \mathop \sum \limits_{s = r}^T {\tau _{rs}}\left( {{w_{idt}} \times {d_r} \times {f_{st}}} \right) + \ldots \ldots $$

(4) $$ + \mathop \sum \limits_{r = q}^T {\delta _{rs}}\left( {{d_r} \times t} \right) + {{\gamma }_t} + {{\delta }_{\mathrm{d}}} + {\phi _c} + {X_{idt}}\lambda + {u_{{\mathrm{idt}}}}$$

We assess the statistical significance of the interaction terms between cohort-specific indicators and time (pre-RBF) to test the assumption of parallel trends. The null hypothesis posits that the coefficients on these interaction terms are jointly zero, suggesting that pre-policy trends for the exposed and non-exposed groups are similar. Failure to reject this hypothesis supports the validity of our ETWFE approach. In all regressions, we include survey probability weights that account for pooling across multiple waves of the Zimbabwe DHS, the sampling design and survey non-response to ensure the representativeness of our estimates.

Our analysis confirms the assumption of parallel trends for most maternal health inequality outcomes, except asset wealth-related inequality in delivery by caesarean-section, which may require further investigation. These findings support the validity of our ETWFE estimates. To further validate the robustness of our findings, we conducted several placebo checks, which involve testing for effects in periods or on outcomes where no effects are expected if our results reflect true programme impacts. The results indicate no statistically significant effects of placebo treatment start years on wealth-related inequalities in prenatal care, delivery services and prenatal care quality outcomes. Additionally, we found no significant effects of RBF on outcomes not targeted by the programme. These findings suggest that our primary results are not driven by spurious associations or pre-existing trends. The results from the parallel trends assumption tests and placebo checks are available as Supplementary materials.

## Results

### RBF and asset wealth-related inequalities in prenatal and delivery care services

Table 2 presents the results from the ETWFE model estimated using Equation (2). The association between the RBF programme and asset wealth-related inequalities in maternal health outcomes is significant. Specifically, the programme is associated with a decrease in disparities in prenatal care access, as indicated by reductions in inequality for completing at least four prenatal care visits (−0.026, *P* < 0.01) and first-trimester prenatal care (−0.033, *P* < 0.01). These negative coefficients suggest that inequalities in these areas declined more rapidly in RBF districts than in non-RBF districts, indicating that the RBF programme may have contributed to improved access to timely and adequate prenatal care services for women of lower socio-economic status compared with their wealthier counterparts. Regarding delivery services, the results show significant associations between the RBF programme and decreased wealth-related disparities for delivery by caesarean section (−0.028, *P* < 0.01) and family planning (−0.033, *P* < 0.01). These findings suggest that inequalities in these maternal health services also declined more rapidly in RBF districts, indicating that the programme may have facilitated greater access to necessary surgical childbirth interventions and family planning services for women from lower socio-economic groups. Conversely, the non-significant associations for general prenatal care visits (−0.005, *P* = 0.640), facility birth delivery (−0.005, *P* = 0.499) and professional delivery assistance (0.009, *P* = 0.225) suggest areas where the RBF programme did not significantly impact asset wealth-related inequalities.

**Table 2.** Estimation results from ETWFE for overall ATT on wealth-related inequality in prenatal care and delivery service outcomes in Zimbabwe

	Prenatal care visits	Four or more prenatal care visits	First trimester prenatal care	Facility birth delivery	Professional delivery assistance	Delivery by caesarean-section	Family planning
ATT estimate	−0.005	−0.026	−0.033	−0.005	0.009	−0.028	−0.033
Standard errors	0.011	0.008	0.007	0.008	0.008	0.005	0.005
*P*-value	0.640	0.002	0.000	0.499	0.225	0.000	0.000

Notes: This table presents the aggregated overall ATT coefficients derived from the ETWFE estimator, using wealth-related inequality in prenatal care and delivery service outcomes in Zimbabwe as the dependent variables. The model includes interaction terms for treatment cohorts and post-treatment periods, controlling for the woman’s age at first birth, age at survey date, female household head, minimum distance to a health facility, presence of children <5 years in the household, employment in agriculture, pregnancy wantedness status, urban residence, birth-year fixed effects, survey-year fixed effects, region fixed effects and a dummy variable for exposure to the 1980 school reform in Zimbabwe. We incorporate region and cluster-specific fixed effects to account for unobserved heterogeneity. All regressions are weighted using survey probability weights that have been adjusted for pooling across multiple rounds of the Zimbabwe DHS, the sampling design and survey non-response, ensuring the representativeness of estimates. Standard errors are robust and clustered at the district level.

### RBF and asset wealth-related inequalities in prenatal care quality outcomes

Table 3 provides the results from the ETWFE model estimated using Equation (2) for asset wealth-related inequalities in prenatal care quality outcomes. The RBF programme significantly reduces disparities in several key prenatal care quality measures. Specifically, the programme is associated with decreases in inequalities in the prenatal care quality index (−0.040, *P* < 0.01), which is a composite index made up of key components such as blood pressure (BP) checks, urine checks and tetanus toxoid (TT) vaccinations. If the individual measures of quality are assessed, the results show that there are significant reductions in disparities for BP checks (−0.024, *P* < 0.01) and TT vaccinations (−0.060, *P* < 0.01), demonstrating the programme’s effectiveness in improving the quality of prenatal care for women from lower socio-economic backgrounds. Interestingly, the results for urine sample checks show an increase in inequality (0.019, *P* < 0.01), suggesting that this measure did not benefit uniformly across different socio-economic groups. The modest reduction in asset wealth-related inequality for blood sample checks (−0.009, *P* < 0.05) also indicates a positive, albeit more negligible, effect. Conversely, the effect on asset wealth-related inequalities in the provision of iron tablets (−0.007, *P* = 0.101) shows no significant impact, suggesting that the RBF programme may not have effectively addressed this aspect. These findings indicate that while the RBF programme significantly improves several aspects of prenatal care quality, its effectiveness varies across different maternal health measures.

**Table 3.** Estimation results from ETWFE for overall ATT on wealth-related inequality in prenatal care quality outcomes in Zimbabwe

	Prenatal care quality index	BP check	Urine sample check	Blood sample check	TT vaccinations	Iron tablets
ATT estimate	−0.040	−0.024	0.019	−0.009	−0.060	−0.007
Standard errors	0.004	0.003	0.005	0.004	0.005	0.004
*P*-value	0.000	0.000	0.000	0.032	0.000	0.101

Notes: This table presents the aggregated overall ATT coefficients derived from the ETWFE estimator, using wealth-related inequality in prenatal care quality outcomes in Zimbabwe as the dependent variables. The model includes interaction terms for treatment cohorts and post-treatment periods, controlling for the woman’s age at first birth, age at survey date, female household head, minimum distance to a health facility, presence of children <5 years in the household, employment in agriculture, pregnancy wantedness status, urban residence, birth-year fixed effects, survey-year fixed effects, region fixed effects and a dummy variable for exposure to the 1980 school reform in Zimbabwe. We incorporate region and cluster fixed effects to account for unobserved heterogeneity. All regressions are weighted using survey probability weights that have been adjusted for pooling across multiple rounds of the Zimbabwe DHS, the sampling design and survey non-response, ensuring the representativeness of estimates. Standard errors are robust and clustered at the district level.

### RBF and absolute inequality in prenatal care and delivery services

To strengthen the robustness of our estimates, we adopt an alternative definition of maternal health inequality using the SII. The SII is a widely recognized measure of absolute inequality in both epidemiology and economics literature, expressing health inequality as the difference in rates between the highest and lowest socio-economic status groups (Mackenbach and Kunst, 1997; Barros *et al*., 2013; Moreno-Betancur *et al*., 2015). We calculated the SII for binary health outcomes using logistic regression, regressing the health outcome of interest against the midpoint value of household asset wealth quintiles (Barros *et al*., 2013).

Table 4 presents the results from the ETWFE model for the SII in prenatal care and delivery service outcomes. The RBF programme is significantly associated with reductions in absolute inequality across several key measures. Specifically, the programme is associated with decreases in the SII for prenatal care visits (−0.058, *P* < 0.01), four or more prenatal care visits (−0.189, *P* < 0.01) and first-trimester prenatal care (−0.088, *P* < 0.01). These negative coefficients suggest that the RBF programme is associated with reduced absolute inequalities in access to prenatal care, indicating enhanced access for women from lower socio-economic backgrounds. For delivery services, the programme is associated with significant reductions in the SII for facility birth delivery (−0.072, *P* < 0.01), professional delivery assistance (−0.057, *P* < 0.01) and delivery by caesarean section (−0.072, *P* < 0.01). These findings highlight the programme’s potential effectiveness in improving equitable access to safer delivery options and skilled birth attendance, particularly benefiting women from disadvantaged socio-economic groups. However, the results for family planning (0.005, *P* = 0.431) show no significant association, indicating that the RBF programme does not significantly influence absolute inequality in this outcome.

**Table 4.** Estimation results from ETWFE for overall ATT on the SII in prenatal care and delivery service outcomes in Zimbabwe

	Prenatal care visits	Four or more prenatal care visits	First trimester prenatal care	Facility birth delivery	Professional delivery assistance	Delivery by caesarean-section	Family planning
ATT estimate	−0.058	−0.189	−0.088	−0.072	−0.057	−0.072	0.005
Standard errors	0.007	0.015	0.011	0.010	0.009	0.008	0.006
*P*-value	0.000	0.000	0.000	0.000	0.000	0.000	0.431

Notes: This table presents the aggregated overall ATT coefficients derived from the ETWFE estimator, using the SII in prenatal care and delivery service outcomes in Zimbabwe as the dependent variables. The model includes interaction terms for treatment cohorts and post-treatment periods, controlling for the woman’s age at first birth, age at survey date, female household head, minimum distance to a health facility, presence of children <5 years in the household, employment in agriculture, pregnancy wantedness status, urban residence, birth-year fixed effects, survey-year fixed effects, region fixed effects and a dummy variable for exposure to the 1980 school reform in Zimbabwe. We incorporate region and cluster fixed effects to account for unobserved heterogeneity. All regressions are weighted using survey probability weights that have been adjusted for pooling across multiple rounds of the Zimbabwe DHS, the sampling design and survey non-response, ensuring the representativeness of estimates. Standard errors are robust and clustered at the district level.

### RBF and absolute inequality in prenatal care quality outcomes

Table 5 provides the results from the ETWFE model for the SII in prenatal care quality outcomes. The RBF programme is significantly associated with reductions in absolute inequality in several prenatal care quality components. Specifically, the programme is associated with decreases in the SII for the overall prenatal care quality index (−0.036, *P* < 0.01), which comprises key components such as BP checks and blood sample checks. Notably, the results show significant reductions in disparities for specific prenatal care quality components, particularly in BP checks (−0.111, *P* < 0.01) and blood sample checks (−0.045, *P* < 0.01). These findings suggest that the RBF programme improves equitable access to high-quality prenatal care services, benefiting women from lower socio-economic backgrounds. However, the results for the urine sample checks (0.039, *P* < 0.01) show a significant positive association in RBF districts, indicating an increase in absolute inequality, with access to this service appearing to favour women from relatively wealthier families. This variability suggests that the programme’s impact on prenatal care quality is not uniform across all measures. The SII for TT vaccinations (−0.010, *P* = 0.166) does not show a significant association, indicating that the RBF programme does not significantly influence absolute inequality in this aspect of prenatal care quality. The SII for iron tablet provision significantly decreases by 0.028 (*P* < 0.01), indicating a substantive reduction in absolute inequality associated with this maternal health service.

**Table 5.** Estimation results from ETWFE for overall ATT on the SII in prenatal care quality outcomes in Zimbabwe

	Prenatal care quality index	BP check	Urine sample check	Blood sample check	TT vaccinations	Iron tablets
ATT estimate	−0.036	−0.111	0.039	−0.045	−0.010	−0.028
Standard errors	0.006	0.011	0.010	0.012	0.007	0.010
*P*-value	0.000	0.000	0.000	0.000	0.166	0.004

Notes: This table presents the aggregated overall ATT coefficients derived from the ETWFE estimator, using the SII in prenatal care quality outcomes in Zimbabwe as the dependent variables. The model includes interaction terms for treatment cohorts and post-treatment periods, controlling for the woman’s age at first birth, age at survey date, female household head, minimum distance to a health facility, presence of children <5 years in the household, employment in agriculture, pregnancy wantedness status, urban residence, birth-year fixed effects, survey-year fixed effects, region fixed effects and a dummy variable for exposure to the 1980 school reform in Zimbabwe. We incorporate region and cluster-specific fixed effects to account for unobserved heterogeneity. All regressions are weighted using survey probability weights that have been adjusted for pooling across multiple rounds of the Zimbabwe DHS, the sampling design and survey non-response, ensuring the representativeness of estimates. Standard errors are robust and clustered at the district level.

## Discussion

This study examines the association between the RBF programme and asset wealth-related inequalities in maternal health outcomes in Zimbabwe, focusing on disparities in prenatal and delivery care services across socio-economic groups. We assess the programme’s potential to reduce these disparities and promote equitable access to healthcare in resource-constrained settings. Using nationally representative data from the Zimbabwe DHS, combined with geo-linked health facility data, we estimate ETWFE models that leverage geographic variation and the staggered rollout of the RBF programme to evaluate its impact on healthcare access.

Our findings reveal significant reductions in both relative and absolute health inequalities, particularly in the frequency and timing of prenatal care, delivery by caesarean-section and family planning. These results highlight significant progress in making healthcare more accessible, particularly for women from lower socio-economic status groups. These findings have important implications for health policy, emphasizing the necessity of integrating equity considerations into health system reforms to ensure that improvements in access are distributed fairly across all population groups. Moreover, our results align with recent studies, such as Mwase *et al*. (2022), which demonstrate that combining RBF with equity-focused measures can result in greater fairness in the utilization of maternal healthcare services. Building on this analysis, Makate (2024) presents robust evidence from Zimbabwe, demonstrating that RBF not only improves maternal health outcomes but also significantly reduces IOP in critical areas such as prenatal care, early prenatal care, facility births and professional delivery assistance. While focused specifically on IOP, these findings underscore the broader capacity of RBF to enhance equitable access to maternal healthcare, particularly in low-income settings where disparities are more entrenched.

However, Lohmann *et al*. (2022) highlight the complexity of achieving equity through performance-based financing, underscoring the importance of contextual, design and implementation factors. Others like Ridde *et al*. (2018) emphasize the necessity of incorporating equity measures in RBF programmes to ensure that they effectively address disparities. Additionally, Priedeman Skiles *et al*. (2013) provide evidence from Rwanda showing that while RBF programmes increased overall service use, they did not specifically target the poorest populations. This observation suggests that additional equity-focused measures are needed to close the gap in service utilization. Our findings contribute to this discourse by demonstrating that, when appropriately designed and implemented, RBF can indeed reduce inequalities in maternal health outcomes, particularly among disadvantaged groups. These results align with the existing literature that highlights the positive effects of RBF on access to maternal health services, although it has not always explicitly addressed the extent of inequalities in these outcomes (Basinga *et al*., 2011; World Bank, 2016; Fichera *et al*., 2021; de Walque *et al*., 2022).

Our study suggests several policy implications for enhancing the effectiveness of RBF programmes in reducing inequalities in maternal health outcomes. The significant reductions in asset wealth-related inequalities in prenatal care and caesarean-section deliveries underscore the need to improve access in underserved areas. Policymakers should prioritize expanding and strategically placing prenatal and delivery services in underserved areas, such as Matabeleland North and Mashonaland Central, to ensure equitable access to essential services. Reports highlight that women in these regions face substantial challenges accessing maternal health services due to distance and resource constraints (UNICEF, 2021; Webb *et al*., 2021).

Incorporating and strengthening targeted equity measures within RBF frameworks is also important. Our findings indicate that the RBF programme has been effective in reducing health inequalities, particularly for the poorest populations. To build on this success, programmes should align financial incentives with equity-focused outcomes. Strengthening these measures will promote inclusive healthcare access and ensure that the most disadvantaged groups benefit significantly from improved maternal health services.

Continuous monitoring and evaluation of RBF programmes are essential. The variability in the impact of RBF on different maternal health outcomes underscores the critical need for ongoing monitoring and evaluation. Health policy planners should establish robust systems to regularly assess the effectiveness of these programmes in reducing health inequalities. Such systems will enable timely adjustments to strategies and interventions, ensuring that the programmes remain responsive to the evolving needs of the population and continue to promote equitable healthcare access effectively.

This study provides important insights into the impact of the RBF programme on asset wealth-related inequalities in maternal health outcomes in Zimbabwe, but it is not without limitations. First, reliance on cross-sectional DHS data limits our ability to capture long-term changes and trends, and studies of this nature would benefit from longitudinal datasets. Second, although methodologically sound, our geo-linking of household data to health facilities may be affected by the intentional displacement of geographic coordinates by DHS to preserve privacy and confidentiality, which could impact the accuracy of proximity measures (Burgert *et al*., 2013). Unfortunately, this limitation is inherent to the DHS data and cannot be addressed within the scope of our analysis. Third, potential spillover effects, where RBF districts might influence neighbouring non-RBF districts, could complicate the interpretation of RBF’s true impact. We attempted to minimize these effects by controlling for regional, district and cluster fixed effects. Despite our best efforts, some residual confounding may persist. Finally, asset-based measures may not comprehensively capture household equity due to the slow-changing nature of household assets and the persistence of high-value assets in some households. However, asset-based measures remain widely used in low-income countries due to their practicality and relative ease of collection (Sahn and Stifel, 2003; Booysen *et al*., 2008). Despite these considerations, our study contributes significantly to understanding the role of RBF in reducing health inequalities in resource-limited settings, emphasizing the need for continued, in-depth research and empirical evidence in this area.

## Conclusion

This study adds a novel perspective to the literature on RBF programmes in low-income countries by examining their impact on asset wealth-related inequalities in maternal health outcomes in Zimbabwe. Our analysis reveals significant reductions in disparities related to prenatal care frequency and timing, as well as in caesarean-section deliveries, highlighting the potential of RBF programmes to improve equitable healthcare access, particularly for women from lower socio-economic backgrounds. We recommend integrating targeted equity measures within RBF frameworks, prioritizing the strategic expansion of maternal health services in underserved regions, and establishing robust monitoring and evaluation systems to ensure these programmes effectively reduce health disparities. Future research should explore the long-term impacts of RBF programmes on asset wealth-related inequality in maternal and child health outcomes beyond the initial implementation period, providing insights into their sustainability and effectiveness over time. Additionally, cross-country comparative studies could illuminate contextual factors that influence how RBF programmes address disparities in maternal health outcomes, aiding policymakers in tailoring interventions to specific regional needs.

## Supplementary Material

czae080_Supp

## Footnotes

1. Researchers interested in using this publicly available dataset can formally request access, subject to authorization from the MEASURE DHS team. Access to the DHS data for Zimbabwe requires formal authorisation, and detailed steps for securing the data can be found at: https://dhsprogram.com/data/Access-Instructions.cfm.

## Reflexivity statement

As senior lecturers in economics, the authors share a common foundation in economic analysis and policy evaluation, albeit with distinct areas of specialization and geographical focus. The lead author (M.M.) is a health economist with deep roots in Zimbabwe, a low–middle income country (LMIC) that is central to this study. His expertise spans economic evaluation, policy analysis and applied econometrics, with a particular emphasis on global health issues and health systems improvements, not only in LMICs but also in high-income settings like Australia. This diverse experience enriches our understanding of the complexities of RBF in maternal health. The co-author (N.M.) brings a broad perspective from development economics, macroeconomics, policy analysis, and monetary economics. Together, our collective expertise in conducting quantitative research in LMICs underpins a comprehensive analysis of RBF’s impact on socio-economic-related inequalities in maternal health outcomes in Zimbabwe. Our backgrounds, combined with our academic and personal experiences, inform our interpretation of the data and findings, guiding us to contribute thoughtfully to the discourse on health financing reforms in LMICs.
