# Supplementary material for: Closing the gap? Results-based financing and socio-economic-related inequalities in maternal health outcomes in Zimbabwe
Source: Health Policy Plan. 2024 Aug 24;39(10):1022–31. doi: 10.1093/heapol/czae080 (PMC11562138; doi:10.1093/heapol/czae080)
Supplement: czae080_Supp [file czae080_supp.zip › Supplementary Materials R3.docx]

**Closing the gap? Results-based financing and socioeconomic-related inequalities in maternal health outcomes in Zimbabwe.**

SUPPLEMENTARY MATERIALS

19 August 2024

1. **List of variables and definitions**

- **CI prenatal care visits**: The corrected concentration index for the number of antenatal care visits the respondent completed during the most recent pregnancy.
- **CI prenatal care visits (4+)**: The corrected concentration index for completing four or more antenatal care visits during the most recent pregnancy.
- **CI first trimester prenatal**: The corrected concentration index for first trimester antenatal care during the most recent pregnancy.
- **CI facility delivery**: The corrected concentration index for delivery in a health facility for the most recent pregnancy.
- **CI professional delivery**: The corrected concentration index for delivery assistance by a qualified health professional or the most recent pregnancy.
- **CI c-section delivery**: The corrected concentration index for delivery by caesarean section for the most recent pregnancy.
- **CI family planning**: The corrected concentration index for knowledge or use of a modern family planning method.
- **CI prenatal care quality index**: The corrected concentration index for the quality of prenatal care index for the most recent pregnancy. The prenatal care quality index is an additive index found by adding dummy indicator variables for having received the following prenatal care components during pregnancy: blood pressure check, urine sample collected, blood sample checked, iron tablets received, and tetanus toxoid vaccinations received.
  - **CI blood pressure check**: The corrected concentration index for blood pressure check during antenatal care for the most recent pregnancy.
  - **CI urine sample check**: The corrected concentration index for urine sample check during antenatal care for the most recent pregnancy.
  - **CI blood sample check**: The corrected concentration index for blood sample check during antenatal care for the most recent pregnancy.
  - **CI blood iron tablets**: The corrected concentration index for receipt of iron tablets during antenatal care for the most recent pregnancy.
- **Post**: A dummy variable that equals one if the DHS year is 2015 (after RBF) and zero if the DHS year is 2010.
- **Cohort 2011**: A dummy variable that equals one if the RBF treatment start year is 2011 and zero otherwise.
- **Cohort 2012**: A dummy variable that equals one if the RBF treatment start year is 2012 and zero otherwise.
- **Cohort 2014**: A dummy variable that equals one if the RBF treatment start year is 2014 and zero otherwise.
- **Treatment**: A dummy variable that equals one if the respondent was exposed to the RBF program based on the estimated conception date and the RBF rollout phase, and zero otherwise. Specifically, the variable equals one if the respondent conceived after July 2011 and lived in a district that adopted RBF in the pilot phase, conceived after March 2012 and lived in a district that adopted RBF in the mid-adopter phase, or conceived after July 2014 and lived in a district that adopted RBF in the late-adopter phase.
- **Age**: The age of the respondent (in years) at the time of the survey. This is defined as the difference between the birth year and the DHS year.
- **Age at first birth**: This is the age of the respondent (in years) at the time of her first childbirth. This is defined as the difference between the birth year and the birth year of the first child.
- **Years of schooling**: The number of years of completed schooling of the respondent as observed at the time of survey.
- **Completed secondary school**: A dummy variable that equals one if the respondent has completed secondary school at the time of the survey and zero otherwise.
- **Literate**: A dummy variable equals one if the respondent can read a whole sentence and zero otherwise.
- **Apostolic church membership:** A dummy variable that equals one if the respondent is a member of or attends the Apostolic church and zero otherwise.
- **Health insurance:** A dummy variable that equals one if the respondent has health insurance and zero otherwise.
- **Asset quintile 1:** A dummy variable that equals one if the respondent’s family is classified as belonging to the poorest asset wealth quintile 1 and zero otherwise.
- **Asset quintile 2:** A dummy variable that equals one if the respondent’s family is classified as belonging to the poor asset wealth quintile 2 and zero otherwise.
- **Asset quintile 3:** A dummy variable that equals one if the respondent’s family is classified as belonging to the average asset wealth quintile 3 and zero otherwise.
- **Asset quintile 4:** A dummy variable that equals one if the respondent’s family is classified as belonging to the rich asset wealth quintile 4 and zero otherwise.
- **Asset quintile 5:** A dummy variable that equals one if the respondent’s family is classified as belonging to the richest asset wealth quintile 5 and zero otherwise.
- **Low asset wealth:** A dummy variable set to one for respondents whose families fall within the two lowest asset wealth quintiles (quintile 1 and 2, poorest and poorer), and zero otherwise.
- **High asset wealth:** A dummy variable set to one for respondents whose families fall within the two highest asset wealth quintiles (quintile 4 and 5, richer and richest), and zero otherwise.
- **Husband’s years of schooling:** The number of years of completed schooling of the respondent’s husband or partner as observed at the time of survey.
- **Woman working:** A dummy variable that equals one if the respondent is currently working and zero otherwise.
- **Urban residence:** A dummy variable that equals one if the respondent lives in an urban area and zero otherwise.
- **Distance to nearest health facility:**
  - **0-5 km:** A dummy variable that equals one if the respondent lives within 5 kilometres from a health facility and zero otherwise.
  - **5-10 km:** A dummy variable that equals one if the respondent lives 5-10 kilometres away from the nearest health facility and zero otherwise.
  - **10+ km:** A dummy variable that equals one if the respondent lives more than 10 kilometres away from a health facility and zero otherwise.
- **Region:** Respondent’s region of residence (Manicaland, Mashonaland Central, Mashonaland East, Mashonaland West, Matabeleland North, Matabeleland South, Midlands, Masvingo, Harare, and Bulawayo). For all regressions, we include dummy variables for region/province or region fixed effects.

| Table A1: Results of the parallel trends analysis for wealth-related inequalities in prenatal care and delivery services outcomes | | |
| --- | --- | --- |
| **Dep. Var: Concentration index** | **F statistic** | **P-value** |
| Prenatal care visits | 0.813 | 0.492 |
| Prenatal care (4 or more visits) | 1.114 | 0.351 |
| First trimester prenatal care | 2.114 | 0.108 |
| Facility birth delivery | 2.330 | 0.084 |
| Professional delivery assistance | 2.399 | 0.077 |
| Delivery by C-section | 3.607 | 0.019 |
| Family planning | 2.327 | 0.084 |

Notes**:** This table presents the results from testing the parallel trends assumption, essential for the extended two-way fixed effects analysis assessing the impact of various prenatal care and delivery services outcomes. The regression models include control variables such as birth year, month of conception, child mobility, DHS survey year, urban residence indicator, age, household size, availability of improved toilet and piped water facilities, regional fixed effects, and district number. Standard errors are robust and clustered at the district level. F-test [p-value] reports the joint significance test of the interaction coefficients.

| Table A2: Results of the parallel trend analysis for wealth-related inequality in prenatal care quality outcomes | | |
| --- | --- | --- |
| **Dep. Var: Concentration index** | **F statistic** | **P-value** |
| Prenatal care quality index | 0.935 | 0.430 |
| Blood pressure check | 1.626 | 0.193 |
| Urine Sample check | 1.622 | 0.194 |
| Blood sample check | 0.736 | 0.535 |
| Tetanus toxoid vaccinations | 1.030 | 0.386 |
| Iron tablets | 1.596 | 0.200 |

Notes**:** This table presents the results from testing the parallel trends assumption, essential for the extended two-way fixed effects analysis assessing wealth-related inequalities in prenatal care quality outcomes. The regression models include control variables such as birth year, month of conception, child mobility, DHS survey year, urban residence indicator, age, household size, availability of improved toilet and piped water facilities, regional fixed effects, and district number. Standard errors are robust and clustered at the district level. F-test [p-value] reports the joint significance test of the interaction coefficients.

| Table A3: Placebo tests: Estimation results from ETWFE for overall ATT on wealth-related inequality in prenatal care and delivery services outcomes in Zimbabwe. | | | | | | | |
| --- | --- | --- | --- | --- | --- | --- | --- |
|  | Prenatal  care visits | 4 or more  prenatal care visits | First trimester  prenatal care | Facility  birth delivery | Professional  delivery assistance | Delivery  by c-section | Family  planning |
| ATT estimate | -0.003 | -0.004 | -0.003 | -0.001 | -0.001 | 0.001 | -0.002 |
| Standard errors | 0.004 | 0.003 | 0.002 | 0.002 | 0.002 | 0.001 | 0.002 |
| P-value | 0.379 | 0.153 | 0.136 | 0.724 | 0.707 | 0.582 | 0.300 |

Notes: This table presents the aggregated overall ATT coefficients derived from the extended two-way fixed effects (ETWFE) estimator using fictitious treatment start years. The placebo test was conducted by assigning random treatment start years (2001, 2002, and 2004) to districts, simulating a treatment start 10 years prior to the actual intervention. All regressions are weighted using survey probability weights that have been adjusted for pooling across multiple rounds of the Zimbabwe DHS, the sampling design, and survey non-response, ensuring the representativeness of estimates. Standard errors are clustered at the district level.

| Table A4: Placebo tests: Estimation results from ETWFE for overall ATT on wealth-related inequality in prenatal care quality outcomes in Zimbabwe. | | | | | | |
| --- | --- | --- | --- | --- | --- | --- |
|  | Prenatal  care quality index | Blood  pressure check | Urine  Sample check | Blood  sample check | Tetanus  toxoid vaccinations | Iron  tablets |
| ATT estimate | -0.001 | 0.000 | 0.001 | 0.002 | -0.001 | -0.001 |
| Standard errors | 0.002 | 0.001 | 0.002 | 0.002 | 0.002 | 0.002 |
| P-value | 0.641 | 0.740 | 0.642 | 0.283 | 0.618 | 0.647 |

Notes: This table presents the aggregated overall ATT coefficients derived from the extended two-way fixed effects estimator using fictitious treatment start years. The placebo test was conducted by assigning random treatment start years (2001, 2002, and 2004) to districts, simulating a treatment start 10 years prior to the actual intervention. All regressions are weighted using survey probability weights that have been adjusted for pooling across multiple rounds of the Zimbabwe DHS, the sampling design, and survey non-response, ensuring the representativeness of estimates. Standard errors are clustered at the district level.

| Table A5: Placebo tests: Estimation results from ETWFE for overall ATT on other outcomes not targeted by the RBF in Zimbabwe. | | | | | | |
| --- | --- | --- | --- | --- | --- | --- |
|  | Years  of education | Primary  schooling | Secondary  education | Age  at first birth | Household  size | Employed  Partner/husband |
| ATT estimate | 0.017 | 0.012 | -0.022 | -0.009 | 0.213 | -0.040 |
| Standard errors | 0.160 | 0.008 | 0.030 | 0.218 | 0.171 | 0.020 |
| P-value | 0.916 | 0.133 | 0.453 | 0.965 | 0.213 | 0.045 |

**Notes:** This table presents the results of a placebo test, estimating the impact of Results-Based Financing (RBF) on outcomes not targeted by the program in Zimbabwe. The analysis employs the extended two-way fixed effects (ETWFE) model, controlling for several covariates including year of conception fixed effects, conception month fixed effects, region fixed effects, district fixed effects, and cluster fixed effects. Standard errors are clustered at the district level. All regressions are weighted using survey probability weights adjusted for pooling across multiple rounds of the Zimbabwe Demographic and Health Surveys (DHS), accounting for the sampling design and survey non-response to ensure the representativeness of estimates. The placebo outcomes examined include years of education, primary schooling, secondary education, age at first birth, household size, and employment status of partner/husband.

| Table A6: Placebo tests: Estimation results from ETWFE for overall ATT on other outcomes not targeted by the RBF in Zimbabwe. | | | | | | |
| --- | --- | --- | --- | --- | --- | --- |
|  | Frequently  listens to radio | Access  to piped water | Improved  housing | Energy  poor | Age at  first cohabitation | Currently  employed |
| ATT estimate | -0.020 | 0.007 | -0.005 | -0.022 | -0.115 | -0.014 |
| Standard errors | 0.030 | 0.030 | 0.024 | 0.026 | 0.178 | 0.029 |
| P-value | 0.503 | 0.813 | 0.845 | 0.403 | 0.519 | 0.638 |

**Notes:** This table presents the results of a placebo test, estimating the impact of Results-Based Financing (RBF) on outcomes not targeted by the program in Zimbabwe. The analysis employs the extended two-way fixed effects (ETWFE) model, controlling for several covariates including year of conception fixed effects, conception month fixed effects, region fixed effects, district fixed effects, and cluster fixed effects. Standard errors are clustered at the district level. All regressions are weighted using survey probability weights adjusted for pooling across multiple rounds of the Zimbabwe Demographic and Health Surveys (DHS), accounting for the sampling design and survey non-response to ensure the representativeness of estimates. The placebo outcomes examined include years of education, primary schooling, secondary education, age at first birth, household size, and employment status of partner/husband.

Table A7: Two-way fixed effect (TWFE) estimates of the impact of Results-Based Financing on prenatal care and delivery services in Zimbabwe

|  | Prenatal  care visits | | 4 or more  prenatal care visits | | First trimester  prenatal care | | Facility  birth delivery | | Professional  delivery assistance | | Delivery  by c-section | | Family  planning | |
| --- | --- | --- | --- | --- | --- | --- | --- | --- | --- | --- | --- | --- | --- | --- |
| RBF | -0.028^*^ | (0.011) | -0.007 | (0.008) | -0.005 | (0.006) | -0.029^***^ | (0.007) | -0.024^***^ | (0.007) | -0.003 | (0.004) | 0.001 | (0.004) |
| Early RBF - July 2011 | Yes |  | Yes |  | Yes |  | Yes |  | Yes |  | Yes |  | Yes |  |
| Mid RBF - March 2012 | Yes |  | Yes |  | Yes |  | Yes |  | Yes |  | Yes |  | Yes |  |
|  |  |  |  |  |  |  |  |  |  |  |  |  |  |  |
| Region fixed effects | Yes |  | Yes |  | Yes |  | Yes |  | Yes |  | Yes |  | Yes |  |
| District fixed effects | Yes |  | Yes |  | Yes |  | Yes |  | Yes |  | Yes |  | Yes |  |
| Birth year fixed effects | Yes |  | Yes |  | Yes |  | Yes |  | Yes |  | Yes |  | Yes |  |
|  |  |  |  |  |  |  |  |  |  |  |  |  |  |  |
| Observations | 23917 |  | 23917 |  | 23732 |  | 23917 |  | 23917 |  | 22175 |  | 23917 |  |
| Mean of dependent variable | 0.232 |  | 0.128 |  | 0.089 |  | 0.233 |  | 0.180 |  | 0.050 |  | -0.024 |  |

Notes: ***, **, and * denote significance at the 1%, 5%, and 10% levels, respectively. This table presents the impact of the Results-Based Financing program on prenatal care and delivery services in Zimbabwe. Our analysis includes the RBF indicator and dummy variables for the year of RBF introduction in each district. We incorporate dummy variables to account for the staggered introduction of RBF, which occurred in three phases: the early phase starting on July 1, 2011, the mid phase starting on March 1, 2012, and the late phase starting on July 1, 2014. The RBF indicator variable equals one if the conception date was on or after the date of RBF introduction in the district where the mother's closest health facility is located. We include controls for the woman's age at first birth, years of education, household asset wealth quantiles, household headship status, apostolic church membership, number of births in the last five years, urban residence status, household size, distance to the nearest health facility, pregnancy wantedness status, birth year fixed effects, district fixed effects, and region fixed effects. All regressions are weighted using survey probability weights that have been adjusted for pooling across multiple rounds of the Zimbabwe DHS, the sampling design, and survey non-response, ensuring the representativeness of estimates. Standard errors are robust and clustered at the district level.

Table A8: Two-way fixed effect (TWFE) estimates of the impact of Results-Based Financing on prenatal care quality outcomes in Zimbabwe

|  | Prenatal  care quality index | | Blood  pressure check | | Urine  Sample check | | Blood  sample check | | Tetanus  toxoid vaccinations | | Iron  tablets | |
| --- | --- | --- | --- | --- | --- | --- | --- | --- | --- | --- | --- | --- |
| RBF | -0.015^*^ | (0.007) | -0.011^*^ | (0.005) | 0.024^***^ | (0.007) | -0.012 | (0.007) | -0.014^*^ | (0.007) | -0.017^**^ | (0.006) |
| Early RBF - July 2011 | Yes |  | Yes |  | Yes |  | Yes |  | Yes |  | Yes |  |
| Mid RBF - March 2012 | Yes |  | Yes |  | Yes |  | Yes |  | Yes |  | Yes |  |
|  |  |  |  |  |  |  |  |  |  |  |  |  |
| Region fixed effects | Yes |  | Yes |  | Yes |  | Yes |  | Yes |  | Yes |  |
| District fixed effects | Yes |  | Yes |  | Yes |  | Yes |  | Yes |  | Yes |  |
| Birth year fixed effects | Yes |  | Yes |  | Yes |  | Yes |  | Yes |  | Yes |  |
|  |  |  |  |  |  |  |  |  |  |  |  |  |
| Observations | 23917 |  | 23917 |  | 23823 |  | 23917 |  | 23917 |  | 23917 |  |
| Mean of dependent variable | 0.157 |  | 0.090 |  | 0.156 |  | 0.132 |  | 0.056 |  | 0.063 |  |

Notes: ***, **, and * denote significance at the 1%, 5%, and 10% levels, respectively. This table presents the impact of the Results-Based Financing program on prenatal care quality outcomes in Zimbabwe. Our analysis includes the RBF indicator and dummy variables for the year of RBF introduction in each district. We incorporate dummy variables to account for the staggered introduction of RBF, which occurred in three phases: the early phase starting on July 1, 2011, the mid phase starting on March 1, 2012, and the late phase starting on July 1, 2014. The RBF indicator variable equals one if the conception date was on or after the date of RBF introduction in the district where the mother's closest health facility is located. We include controls for the woman's age at first birth, years of education, household asset wealth quantiles, household headship status, apostolic church membership, number of births in the last five years, urban residence status, household size, distance to the nearest health facility, pregnancy wantedness status, birth year fixed effects, district fixed effects, and region fixed effects. All regressions are weighted using survey probability weights that have been adjusted for pooling across multiple rounds of the Zimbabwe DHS, the sampling design, and survey non-response, ensuring the representativeness of estimates. Standard errors are robust and clustered at the district level.

Table A9: Distribution of districts by adoption phase in the Results-Based Financing program in Zimbabwe

| Early adopters | Mid adopters | Late adopters |
| --- | --- | --- |
|  |  |  |
| Marondera, Zvishavane | Beitbridge, Binga, Bubi, Buhera, Chivi, Gokwe North, Gokwe South, Guruve, Hwange, Kariba, Lupane, Makonde, Matobo, Mbire, Murehwa, Mutoko | Bindura, Bikita, Chegutu, Chikomba, Chipinge, Chirumanzu, Chiredzi, Gutu, Gwanda, Hwedza, Insiza, Kwekwe, Makoni, Mangwe, Marondera Rural, Masvingo, Mazowe, Mhondoro-Ngezi, Mount Darwin, Mudzi, Murehwa, Mutare, Mutasa, Mwenezi, Nkayi, Nyanga, Rushinga, Seke, Shamva, Shurugwi, Sanyati, Tsholotsho, Umguza, Umzingwane, UMP (Uzumba-Maramba-Pfungwe), Victoria Falls, Wedza, Zaka, Zvimba, Mutare Urban, Harare, Bulawayo |
